# Deep brain stimulation modulates pallidal and subthalamic neural oscillations in Tourette's syndrome

**DOI:** 10.1002/brb3.1450

**Published:** 2019-10-24

**Authors:** Guan‐Yu Zhu, Xin‐Yi Geng, Rui‐Li Zhang, Ying‐Chuan Chen, Yu‐Ye Liu, Shou‐Yan Wang, Jian‐Guo Zhang

**Affiliations:** ^1^ Department of Neurosurgery Beijing Tiantan Hospital Capital Medical University Beijing China; ^2^ Institute of Science and Technology for Brain‐Inspired Intelligence Fudan University Shanghai China; ^3^ Key Laboratory of Computational Neuroscience and Brain‐Inspired Intelligence (Fudan University) Ministry of Education Shanghai China; ^4^ Department of Functional Neurosurgery Beijing Neurosurgical Institute Capital Medical University Beijing China

**Keywords:** deep brain stimulation, globus pallidus interna, local field potential, subthalamic nucleus, Tourette's syndrome

## Abstract

**Introduction:**

Previous studies found subthalamic nucleus deep brain stimulation (STN‐DBS) has clinical effect on Parkinson's disease, dystonia, and obsessive compulsive disorder. It is noteworthy that only a few studies report the STN‐DBS for Tourette's syndrome (TS). Globus pallidus interna (GPi)‐DBS is the one of the most common targets for TS. So, this paper aims to investigate the neural oscillations in STN and GPi as well as the DBS effect between these two targets in same patients.

**Methods:**

The local field potentials (LFPs) were simultaneously recorded from the bilateral GPi and STN in four patients with TS. The LFPs were decomposed into neural oscillations, and the frequency and time–frequency characteristics of the neural oscillations were analyzed across the conditions of resting, poststimulation, and movement.

**Results:**

No difference of resting LFP was found between the two targets. The poststimulation period spectral power revealed the high beta and gamma oscillations were recovered after GPi‐DBS but remained attenuated after STN‐DBS. The STN beta oscillation has fewer changes during tics than voluntary movement, and the gamma oscillation was elevated when the tics appeared.

**Conclusion:**

The high beta and gamma oscillations in GPi restored after GPi‐DBS, but not STN‐DBS. High beta and gamma oscillations may have physiological function in resisting tics in TS. The cortex compensation effect might be interfered by the STN‐DBS due to the influence on the hyper‐direct pathway but not GPi‐DBS.

## Graphical abstract

Distinct low‐frequency and high beta oscillations exist in the globus pallidus interna and subthalamic nucleus (STN). High beta and gamma oscillations might reflect potential physiological compensation effect in Tourette's syndrome (TS). The STN‐deep brain stimulation might interfere with the physiological compensation effect in TS.

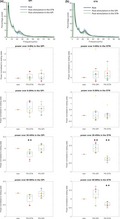

## 1 INTRODUCTION

Tourette's syndrome (TS) is a neurological disorder manifested by motor and vocal or phonic tics. TS is often accompanied by obsessive compulsive disorder, attention‐deficient hyperactive disorder, poor impulse control, and other comorbid behavioral problems (Albin, 2017; Albin & Mink, 2006). Patients with TS have impairments in impulse inhibition which might relate to the reduced inhibitory output of the basal ganglia (Ganos, 2016; Jackson, Draper, Dyke, Pépés, & Jackson, 2015; Jahanshahi & Rothwell, 2017).

Deep brain stimulation (DBS) in the thalamus and basal ganglia has been introduced for the treatment of movement disorders (Zhao, Zhang, & Meng, 2016). The most common targets for TS include the center median thalamic region and the globus pallidus. One study reported a Parkinson's disease (PD) patient who also has a history of TS in whom bilateral subthalamic nucleus (STN)‐DBS improved both PD syndrome and tics. The curative effect of STN‐DBS on dystonia and other hyperkinetic disease has been proved (Schjerling et al., 2013), and the STN‐DBS also shows clinical effect on obsessive compulsive disorder (OCD; Mallet et al., 2008). Overall, STN is a promising target for TS, but the clinical effect and local field potential (LFP) of STN in TS have less been investigated.

Perioperative LFP recordings and intraoperative single‐neuron recordings provide insights into neural activities. The power of low frequency over 1–10 Hz oscillations in the thalamus increases with the occurrence of frequent tics (Shute et al., 2016), and low‐frequency oscillations over 3–12 Hz were found in the pallidus–thalamus circuit, which were correlated with the preoperative motor tic score (Neumann et al., 2018). Moreover, low‐frequency oscillations were found in the thalamus of TS patients with severe compulsive behaviors (Priori et al., 2013). Microelectrode recording in the thalamus of patients with TS further confirmed that the firing patterns were within the low‐frequency range (2.5–8 Hz). DBS at the thalamus tended to attenuate alpha oscillations in the contralateral thalamus and increase theta oscillations in the ipsilateral thalamus in a case study (Bour et al., 2015; Marceglia et al., 2017). Alternatively, in the globus pallidus interna (GPi), attenuated beta oscillation and elevated gamma and high‐frequency oscillations were seen along with frequent tics (Jimenez‐Shahed, Telkes, Viswanathan, & Ince, 2016). Low‐frequency (4–12 Hz) and high‐frequency (>150 Hz) phase–amplitude couplings in the GPi were seen at rest (Ji et al., 2016). So, combining the evidence above, the low‐frequency oscillation is pathological because it related to the clinical scores. The other frequency band might reflect the more complicated cross‐effect when tics appeared.

What are the characteristic oscillatory activities in the GPi and STN of patients with TS? How do STN and GPi‐DBS modulate these activities? This paper aims to answer these questions through an investigation of LFPs recorded simultaneously from the ipsilateral GPi and STN in patients with TS under the conditions of rest, movement, and after high‐frequency stimulation to find explanations for the difference clinical effect of STN and GPi‐DBS on TS.

## 2 MATERIAL AND METHODS

### 2.1 Subjects and surgery

All patients gave written informed consent to participate in this study, which was approved by the local ethics committees of Beijing Tiantan Hospital of Capital Medical University under the principles of the Declaration of Helsinki. Four patients (four males, age 24.8 ± 4 [mean ± *SD*] years, disease duration 18.5 ± 3.4 [mean ± *SD*] years) with TS underwent bilateral GPi and STN implantation of DBS electrodes. Preoperative clinical evaluations using the Yale Global Tic Severity Scale (YGTSS) and the Yale–Brown Obsessive Compulsive Scale (Y‐BOCS) were performed before and after the surgery (Table 1). The patients were followed up for 6 months.

**Table 1:** Clinical summary

Case	Age/Sex	Dominated symptoms	Duration of disease (years)	YGTSS (motor tic/vocal tic/ impairment)	Y‐BOCS (part 1/part 2)	Electrode externalized period (YGTSS/Y‐BOCS)	Final follow‐up Score (YGTSS/Y‐BOCS)	Stimulating contacts	Stimulation parameters at the final follow‐up
1	20/M	Cranial, shoulder, and vocal tics	7	86 (21/15/50)	5 (2/3)	GPI 70/2 STN 72/5	44/5	L‐STN: 0+1−; R‐STN: 0+1−; L‐GPi: 0+2−; R‐GPi: 0+2−	Left: *C* + 3 − 60 μs, 145 Hz, 2.35 V; right: *C* + 3 − 70 μs, 145 Hz, 2.35 V
2	28/M	Cranial and vocal tics	18	64 (13/11/40)	14 (7/8)	GPI 50/10 STN 77/14	35/10	L‐STN: 0+1−; R‐STN: 0+1−; L‐GPi: 0+1−; R‐GPi: 0+1−	Left: C + 2 − 70 μs, 140 Hz, 3.30 V; right: *C* + 2 − 70 μs, 140 Hz, 3.30 V
3	27/M	Tics on limbs and obsession	15	74 (17/7/50)	28 (13/15)	GPI 60/20 STN 60/28	63/18	L‐STN: 0+2−; R‐STN: 0+2−; L‐GPi: 0+1−; R‐GPi: 0+1−	Left: *C* + 2 − 60 μs, 135 Hz, 2.5 V; right: *C* + 2 − 70 μs, 135 Hz, 2.5 V
4	23/M	Tics on limbs, vocal tics, and obsession	17	80 (15/15/50)	26 (10/16)	GPI 58/22 STN 50/26	NA	NA	NA

Abbreviations: GPi, globus pallidus interna; STN, subthalamic nucleus; Y‐BOCS, Yale–Brown obsessive compulsive scale; YGTSS, Yale Global Tic Severity Scale.

All patients underwent stereotactic frame preoperative 3.0T magnetic resonance imaging (MRI). The electrodes were targeted to posterior GPi and the lateral area of STN. The targets and trajectory of the electrode implantation were calculated and determined using the Leksell SurgiPlan station (Elekta). The DBS electrodes were PINS L301 for STN with four platinum–iridium cylindrical surface contacts, and each contact was 1.27 mm in diameter and 1.5 mm in length and separated by 0.5 mm. The electrodes for GPi were PINS L302, which had contacts of the same size but separated by 1.5 mm (PINS). The subjects underwent surgery with local anesthesia to allow for intraoperative microelectrode recording and stimulation tests (Starr, 2002). The placement of DBS electrodes was confirmed by postoperative CT scans.

### 2.2 Paradigm and recording

All patients underwent experiments on the 3–5th day after the surgery. The stimulation was turned off at least 4 hr before the recording. Three channels of bipolar LFPs were recorded from the adjacent four contacts (contact pairs: 0–1, 1–2, and 2–3) of each electrode. The LFPs of patient 1 were amplified, notch filtered to remove 50 Hz line noise, band‐pass filtered over 1–500 Hz using a Digitimer amplifier (model D360; Digitimer Ltd.), and recorded with a sampling frequency of 1,000 Hz using a CED 1401 (Cambridge Electronic Design). The signal was down‐sampled to 500 Hz for further analysis. The LFPs, electromyography (EMG) of symptom‐involved muscles, and forearm flexors of patients 2–4 were amplified and band‐pass filtered over 1–250 Hz using a custom‐developed amplifier and recorded with a sampling frequency of 500 Hz. This amplifier is a wearable eight‐channel wide‐scale electrophysiological monitor with a wireless connection using Wi‐Fi.

The regular clinical test stopped for a whole night before experiments. Three experiments were performed for cases 2–4 on the 3–5th day after DBS electrodes implantation. Case 1 does not perform the voluntary movement test due to the experiment design change. The detailed paradigms were as follows: (a) rest: patients sat at rest without support and the motor/phonic tics were not restricted. The LFPs and EMG of the symptom‐involved muscles were recorded simultaneously for 30 min. During the recording, the patients were asked to avoid voluntary movements. (b) Voluntary movement: patients sat with hands resting on the laps and performed self‐paced wrist‐folding movements for at least 30 trials. The voluntary movements were performed by left and right hands separately. (c) Stimulation test: A pair of contacts was selected for stimulation based on the images and stimulation effects (Table 1). The stimulation was delivered to the active contact with 130 Hz, 60 µs, and amplitudes at 1, 2, and 2.5 V amplitudes. The stimulation was applied bilaterally in the GPi or STN. The duration of stimulation at each amplitude was 180 s with a 180‐s interval between stimulations.

### 2.3 Signal preprocessing

Signal processing and statistical analysis were performed with MATLAB scripts (MathWorks Inc.). The channels with the selected pair of contacts for the stimulation test were used for analysis (Table 1). For the resting state, a continuous 150‐s signal was selected for case 1. Continuous 1,200‐s signals were selected for cases 2–4. Three poststimulation segments form the pair of contacts selected for stimulation were chosen, and the stimulation period was excluded due to the noise and uncertain stimulation effect caused by relatively close distance. LFPs were band‐pass filtered over 3–90 Hz and adaptively band‐stop filtered to reject the 50 Hz line noise. EMG was first high‐pass filtered at 20 Hz and then smoothed with a 5 Hz low‐pass filter.

### 2.4 Spectral analysis and statistical analysis

Power spectral density (PSD) was calculated using Welch's method (Welch, 1967) with a 1‐s sliding window, 0.5‐s overlap, and 2,048 points. The spectral power over 4–8, 8–20, 20–45, and 60–90 Hz frequency bands were integrated to evaluate the sustained effects of stimulation. The 45–60 Hz bands were excluded due to the line noise. For comparison across states at the group level, the integrated oscillatory power during stimulation and poststimulation were normalized to the resting power over the same frequency bands. Paired *T*‐tests were performed between resting and poststimulation in the STN, and resting and poststimulation in the GPi, respectively. The comparison would be considered as significantly different if the *p*‐value is <.00833 (Bonferroni corrected for multiple comparisons across four cases).

### 2.5 Extracting oscillatory modulations by the tics and voluntary movement

The pallidal and subthalamic neural activities modulated by tics and voluntary movement were compared in the time–frequency domain to explore the dynamic changes in multiple frequency ranges. To compare different motor conditions, the spectrograms around the movements were normalized by subtracting and being divided by the resting spectral power at each frequency point. The normalized time–frequency spectrogram was segmented into trials that had a time window over [−1, 1.5] s and was centered at the time of the maximum muscle contraction. The segmented spectrograms were then averaged across trials. As shown in Figure S1, the time of maximum muscle contraction was defined at the peak of the contraction burst. To evaluate the movement‐modulated neural oscillations over frequency bands, the time‐varying dynamics over 4–8, 8–15, 15–30, and 60–75 Hz were integrated.

The subthalamic and pallidal LFPs during movements were recorded simultaneously. We did not calculate the time difference of movement response in the STN and GPi for two reasons. (a) The event‐related desynchronization/event‐related synchronization (ERD/ERS) in the GPi is less clear than in the STN, and they are not consistently found in every trial; (b) It is difficult to define a start time of power change in LFPs due to fluctuations and dynamics. Thus, it is not reliable in methodology to calculate the synchronicity between the nucleus responses to the movements.

## 3 RESULTS

### 3.1 Clinical evaluation

All patients had prodromal symptoms related to motor or vocal tics and comorbidities including obsessions, anxiety, and depression. One patient had stimulation‐induced dyskinesia during STN‐DBS. Patients 1 and 3 had a history of medication treatment with aripiprazole and haloperidol. The YGTSS score is decreased after the STN and GPi‐DBS in two patients, but the YGTSS score is increased after STN‐DBS in one patient in electrode externalized period. The GPi‐DBS also showed a better clinical effect on OCD than STN‐DBS. Postoperative follow‐ups showed that that GPi stimulation effectively improved the symptoms in cases 1–3. Case 4 withdrew all electrodes and hence was not implanted with the pulse generator due to the relative's opposition as clinical efficacy did not meet expectations. All patients preferred the GPi. The average improvements of YGTSS and Y‐BOCS were 41 ± 11% (mean ± *SD*) and 21 ± 19% (mean ± *SD*) of the other three patients at the final follow‐up. All details are shown in Table 1. Note that the Y‐BOCS >25 is considering as dominated OCD syndrome in that patient.

### 3.2 Oscillatory activities at rest

**Figure 1:**
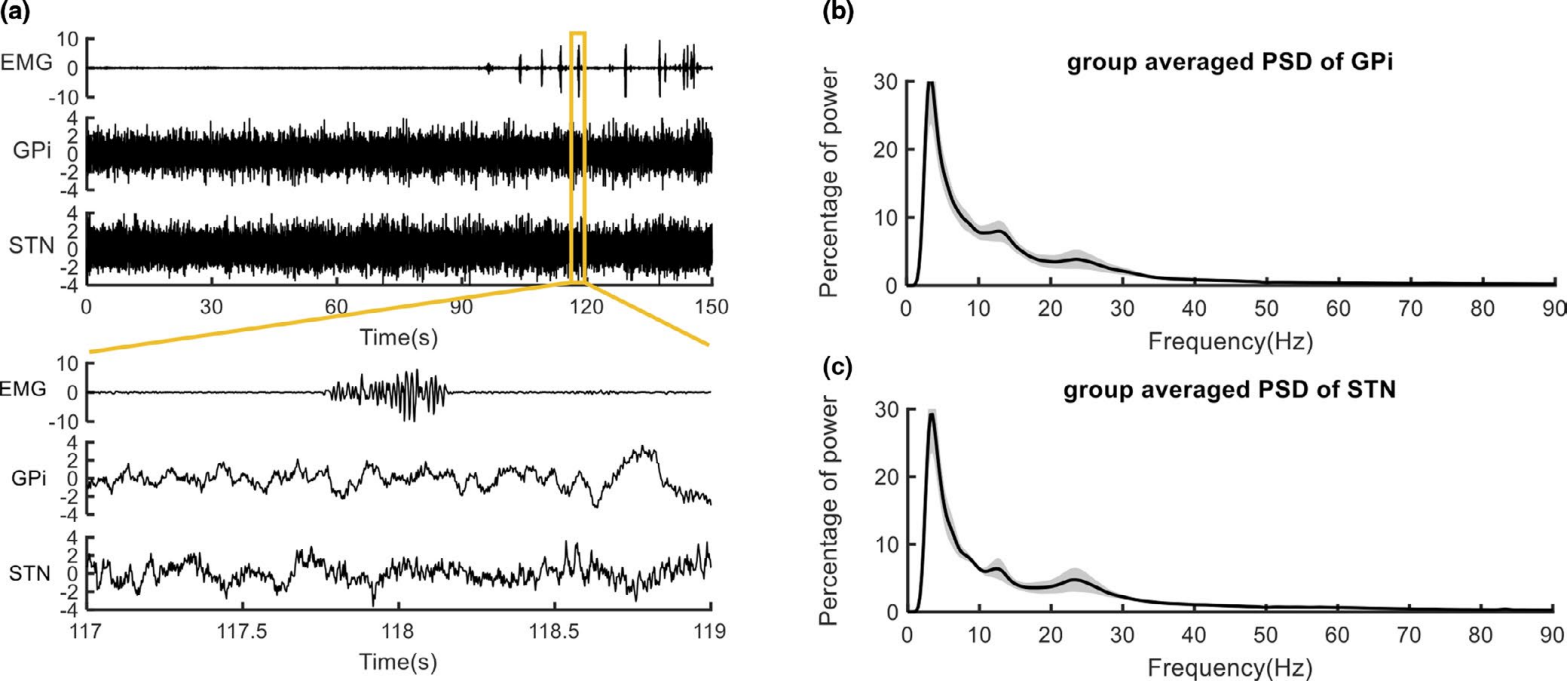
Signals and the power spectral density. The electromyography (EMGs) recorded from the symptom‐involved muscle and local field potentials (LFPs) recorded from ipsilateral globus pallidus interna (GPi) and subthalamic nucleus (STN) in case 2 are shown, respectively, in the time range of 0–150 and 117–119 s in panel (a). The percentage power spectra over 0–90 Hz of LFPs recorded at resting state and averaged across all cases are shown in panels (b and c). The alpha power peaks and high beta power peaks were found in both GPi and STN

Figure 1a shows the surface EMGs of the symptom‐involved muscle and the pallidal and subthalamic LFPs simultaneously recorded from case 2. The percentage power spectra over 0–90 Hz showed distinct power peaks around low frequency and high beta frequency (Figure 1b,c). Specifically, the power peaks over low frequency were found in six GPi with 13 ± 1.5 Hz (mean ± *SD*) and six STN with 12 ± 2.5 Hz (mean ± *SD*). The high beta power peaks were found in five GPi with 26 ± 2 Hz (mean ± *SD*) and six STN with 26 ± 2 Hz (mean ± *SD*; see Figure S2). Moreover, the high beta power peaks were clearer in cases 1 and 3 than in cases 2 and 4. No significant LFP difference was found between the two nuclei at rest.

### 3.3 Poststimulation pallidal and subthalamic oscillations

**Figure 2:**
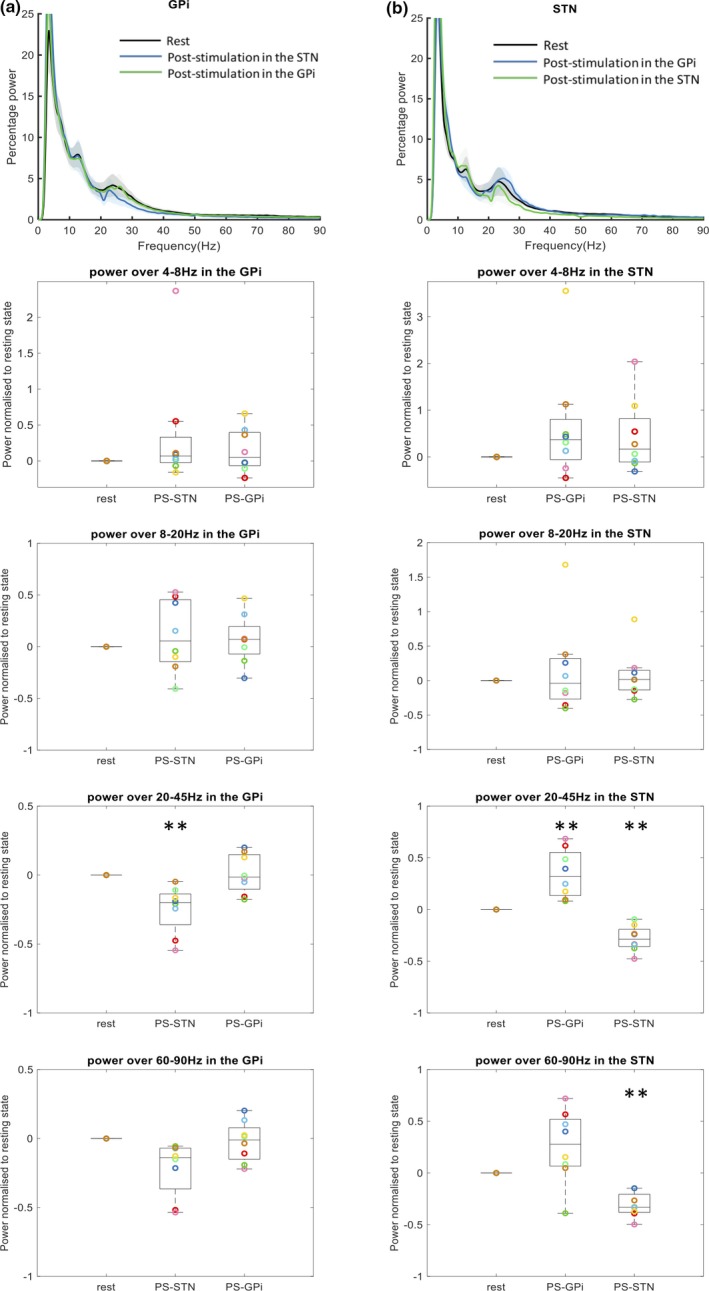
Statistical comparison across resting and poststimulation conditions. Group‐averaged spectral power over 0–90 Hz and oscillatory bands compared across resting, stimulation with 2.5 V in the other nuclei and poststimulation in the other and the same nuclei. Each colored circle represented the integrated frequency power of an individual nucleus. Spectral power over 20–45 and 60–90 Hz in the globus pallidus interna (GPi) was significantly attenuated by subthalamic stimulation and stayed attenuated after the subthalamic stimulation. Spectral power over 20–45 and 60–90 Hz in the subthalamic nucleus (STN) was significantly attenuated by pallidal stimulation and stayed attenuated by the subthalamic stimulation. Moreover, the spectral power over 20–45 Hz significantly increased after the pallidal stimulation compared to the resting state. The statistical analyses were performed with the paired *T*‐test between the resting state and the stimulation/poststimulation states. **p < .008. Abbreviations: PS‐GPi, poststimulation in the GPi; PS‐STN, poststimulation in the STN; S‐GPi, stimulation in the GPi; S‐STN, stimulation in the STN

The stimulation effects to the pallidal and subthalamic oscillations were compared with the poststimulation conditions against the resting condition. As shown in Figure 2, the spectral power over 20–45 Hz in the GPi was significantly attenuated after STN‐DBS compared with rest state (*p* = .005); The spectral power over 60–90 Hz in the GPi after STN‐DBS has descendent tendency, but not significant (*p* = .017). The above two frequency band powers in GPi returned to the resting level after turning off the pallidal stimulation.

The spectral power over 20–45 Hz in STN was elevated after GPi‐DBS (*p* = .004) but attenuated after STN‐DBS (*p* = .0004). The spectral power over 60–90 Hz in STN was attenuated after STN‐DBS (*p* = .0002). The spectral power over 60–90 Hz in STN have an ascendant tendency after GPi‐DBS, but not significant (*p* = .0002). No significant change in the other frequency bands after stimulation in either nucleus can be found in this study (All *p* > .05). All individual LFPs are shown in Figure S2.

### 3.4 Comparison of tic and voluntary movement‐related oscillations

**Figure 3:**
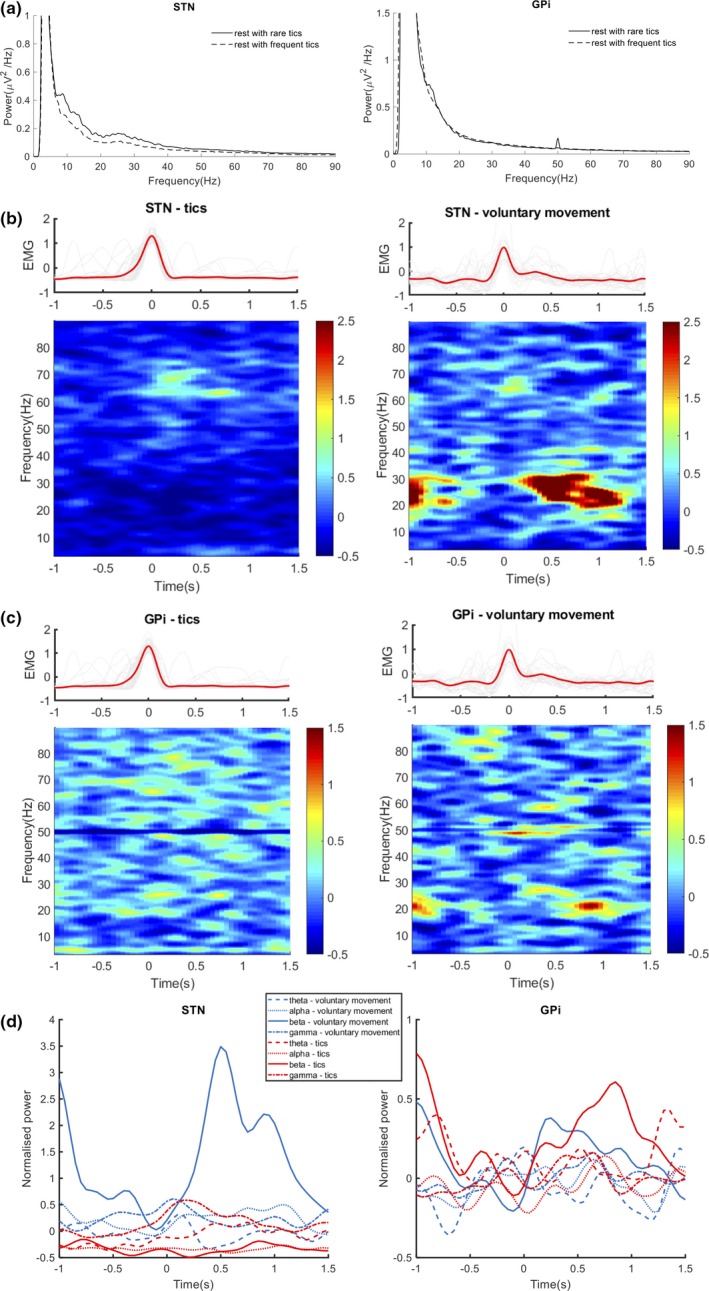
Oscillations modulated by tics and voluntary movement. (a) Power spectra of the local field potential signals in the subthalamic nucleus (STN) and the globus pallidus interna (GPi) at resting state with rare and frequent tics. (b) Trial‐averaged spectrogram of the tics and voluntary movement‐modulated oscillations in the STN and the electromyography (EMG) signals aligned at the time of maximum muscle contraction. (c) Trial‐averaged spectrogram of the tics and voluntary movement‐modulated oscillations in the GPi and the EMG signals aligned at the time of maximum muscle contraction. (d) time‐varying power dynamics of the movement‐modulated oscillations over theta (4–8 Hz), alpha (8–15 Hz), beta (15–30 Hz), and gamma (60–75 Hz) bands. The movement event‐related desynchronization (ERD) and postmovement event‐related synchronization (ERS) over beta band were seen in both nuclei during voluntary movement. The movement ERS over gamma band was seen in the STN during voluntary movement and tics

The pallidal and subthalamic activities related to the involuntary movement of tics were compared with those related to voluntary movement in the time–frequency domain to explore the dynamic changes in multiple frequency ranges. The spectral power over alpha and beta bands was lower at rest with frequent tics compared to rest with rare tics. The movement ERD and the postmovement ERS over the beta (15–30 Hz) band were seen in both the GPi and STN during voluntary movement. The beta ERD/ERS around tics was also found in the GPi but was less clear. The movement ERS over gamma (60–75 Hz) was found in the STN during voluntary movement and tics and was not different between the two conditions. All results are shown in Figure 3.

## 4 DISCUSSION

Distinct low‐frequency (~12 Hz) and high beta (~26 Hz) oscillations exist in the GPi and STN. The STN‐DBS attenuated high beta (20–45 Hz) and gamma (60–90 Hz) oscillations in both the GPi and STN after the stimulation. The GPi‐DBS elevated the high beta oscillations in the STN and do not influence high beta and gamma oscillations in the GPi after the stimulation. Movement‐related beta modulations were found during voluntary movement and tics in both nuclei and were stronger during voluntary movement than during tics. Gamma modulations were found during tics in the STN which might be related to the inhibition compensation effect. With the clinical evaluation that GPi‐DBS showed better clinical effect, we deduced the GPi‐DBS might retain the physiology oscillation while inhibiting the pathological oscillation. On the contrary, the STN might interfere with the cortex compensation effect via the hyper‐direct pathway.

### 4.1 Low‐frequency neural oscillations in TS

Low‐frequency oscillations of approximately 12 Hz were detected in the GPi and the STN in this study. These oscillations over similar frequency bands are found in the thalamus (Bour et al., 2015; Maling, Hashemiyoon, Foote, Okun, & Sanchez, 2012; Marceglia et al., 2010; Zauber, Ahn, Worth, & Rubchinsky, 2014) and GPi (Jimenez‐Shahed et al., 2016; Neumann et al., 2018). The spectral power and burst length of the oscillations are correlated with the severity of tics (Neumann et al., 2018).

The low‐frequency oscillations in the basal ganglia are considered as a hyperactive biomarker in dystonia and in PD with dyskinesia (Chen et al., 2006; Geng et al., 2017). In the thalamus, the power of the oscillations over 1–10 Hz increases with the occurrence of frequent tics (Shute et al., 2016). An increased power of the oscillations over <10 Hz was also observed in the GPi but not in the STN in case 2 at rest with frequent tics. Moreover, the increase in low‐frequency power in the basal ganglia is associated with impulse control disorders in PD (Rodriguez‐Oroz et al., 2010). Similarly, patients with TS have impairments in impulse inhibition and the occurrence of tics reflects the disinhibition of the cortical–basal ganglia network (Almeida et al., 2015; Hashemiyoon, Kuhn, & Visser‐Vandewalle, 2017; Jahanshahi & Rothwell, 2017). The low‐frequency oscillations may contribute to the abnormal hyperactivity and impulsivity in TS.

Deep brain stimulation in the GPi suppresses pallidal low‐frequency oscillations in patients with dystonia and correlates with symptom relief (Barow et al., 2014). In a primate TS model, McCairn et al. showed that the GPi‐DBS disrupts the coherence between neurons and suppresses the synchronized low‐frequency oscillation (McCairn, Iriki, & Isoda, 2015). Excessive stimulation in the STN may induce dyskinesia together with the elevated low‐frequency power (Giannicola et al., 2013). However, after STN or GPi‐DBS, we did not observe the low‐frequency band power was inhibited. The phenomenon might result from the tics do not like rigidity and bradykinesia which is status and DBS have aftereffect on the status, we did not observe the tics in the short poststimulation period.

### 4.2 High beta neural oscillation in TS

High beta oscillations were found in the GPi and STN in this study. Beta oscillation is widely explored in the cortical–basal ganglia network and is considered related to hypoactivity. These oscillations desynchronize before and during the movement onset and reflect effort, velocity, and force of the movement (Anzak et al., 2012; Joundi et al., 2012; Kühn et al., 2004). The power of beta oscillations over 13–35 Hz in the GPi in TS is not correlated with the severity of motor tics but contributes to the prediction of preoperative YGTSS scores (Neumann et al., 2018). Here in this study, the 15–30 Hz beta band power has fewer changes in tics than in voluntary movement, which might also imply that this frequency band is involved in TS. During the occurrence of tics, broadband attenuations over the beta frequency were seen in the STN (Figure 3a). The frequency‐integrated dynamics showed that tics and voluntary movement‐related beta ERD existed in both the GPi and STN, while the degree of power attenuation was lower around tics than around voluntary movement. Considering that facial tics are much smaller in size than a voluntary wrist‐folding movement, this result confirms that beta oscillation is physiologically related to motor behavior and may not be pathological in TS.

Beta oscillation can be subdivided into low beta (<20 Hz) and high beta (>20 Hz) oscillations. In PD, the low beta oscillation is most likely correlated with motor severity and suppressed on levodopa (Little & Brown, 2014; Ray et al., 2008). Alternatively, high beta oscillation is less related to parkinsonian bradykinesia and rarely correlated with the amount of dopamine. High beta oscillation may result from the hyper‐direct pathway (Neumann et al., 2016, 2018; Oswal et al., 2016) and is predominantly driven by the cortex (Hirschmann et al., 2011; Litvak et al., 2010; Williams et al., 2002). The hyper‐direct pathway transfers cortical action in shorter latencies via the STN with high beta activity (Botvinick, Cohen, & Carter, 2004; Brittain et al., 2012) and therefore assists in situations that require rapid stop responses to external and internal triggers (Aron et al., 2007; Eagle et al., 2007). In this regard, relative beta increases are found in multiple cortical areas and STN during reactive and proactive inhibition. Successful impulse control shows higher beta power than unsuccessful impulse control in the STN and downstream nuclei, the GPi (Alegre et al., 2013; Brittain et al., 2012; Brücke et al., 2008; Kühn et al., 2004). Thus, the high beta oscillation in TS may reflect successful impulse control of the internal urge to express tics. Interestingly, more high beta oscillations were seen in cases 1 and 3, which also had fewer tics after the surgery and better improvements during the acute phase, while cases 2 and 4 had fewer high beta oscillations and rapid occurrence of motor and vocal tics. In fact, the high beta oscillation in the basal ganglia might be a compensation that stabilizes the physiological function of the cortical–basal ganglia network (Leventhal et al., 2012; Martin‐Rodríguez & Mir, 2018).

### 4.3 Gamma neural oscillation in TS

Gamma oscillation arises in the subthalamic–globus pallidus feedback loop and occurs during movement. The pallidal and subthalamic gamma synchrony increases during the preparation and execution of voluntary movement in dystonia and PD. The strength of synchronization correlates with the size and force of the movement and arousal state (Anzak et al., 2012; Brücke et al., 2008; Litvak et al., 2012; Tan et al., 2013). Jimenez‐Shahed et al. found that pallidal gamma oscillation over 40–150 Hz enhances during tics (Jimenez‐Shahed et al., 2016). In this study, the subthalamic gamma oscillation can be observed during the onset of tic. In contrast to the movement‐related modulations over the beta band, the wrist‐folding voluntary movement, which is stronger than facial tics, was not accompanied by more gamma synchronization. So, we hypothesize that the high gamma oscillation is also involved in the compensation effect of brain under TS. While the high beta does not change when the tics appeared, this might result from the highly complexity of the modulation effect of the cortex; the high beta might reflect a sustained status maintain which is elevated in TS. The high gamma is related to a sudden inhibition attempt.

### 4.4 Different effects of pallidal stimulation and subthalamic stimulation

In PD, both pallidal and subthalamic stimulations suppress excessive beta synchronization in the stimulated nucleus and alleviate symptoms (Blumenfeld & Brontë‐Stewart, 2015; Bronte‐Stewart et al., 2009). Stimulation in the subthalamic area suppresses pallidal beta oscillation (Brown et al., 2004). Subthalamic stimulation has sustained effects on beta attenuation (Blumenfeld & Brontë‐Stewart, 2015; Eusebio, Cagnan, & Brown, 2012). The parkinsonian motor outcomes from subthalamic and pallidal DBS have been reported as equivalent or different (Rodriguez‐Oroz et al., 2005; Volkmann, 2004). The results suggest that in PD, DBS attenuates the oversynchronized beta oscillation to normal levels and “free up” the neurons to allow task‐related behaviors (Garcia‐Munoz, Carrillo‐Reid, & Arbuthnott, 2010). In contrast, elevated beta oscillation in the cortical–basal ganglia network introduced by stimulation slows the movement (Pogosyan, Gaynor, Eusebio, & Brown, 2009).

The stimulation effects of DBS on the oscillations of basal ganglia in TS have not been widely investigated. The signals in brain is not always “pathology”; it has many physiology functions, like the beta is related to the motor control and gamma is related to the movement speed and vigilance, and the signals help people stop fast to the sudden danger, which is an inhibition effect (Brown & Williams, 2005). Compared to purely inhibition theory, the filter effect of deep brain stimulation is more and more recognized. A moderate function is good (Ashkan, Rogers, Bergman, & Ughratdar, 2017; Chiken & Nambu, 2016; McIntyre, Savasta, Kerkerian‐Le Goff, & Vitek, 2004). In this study, high beta and gamma oscillations restore in the circuit after GPi‐DBS but exhibited sustained suppression after STN‐DBS. Considering the role of high beta oscillation in impulse control, the restored high beta oscillation may reflect a compensation of the cortical–basal ganglia network in stabilizing the physiological functions and inhibiting the tics. This finding suggests that pallidal stimulation may preserve the physiological high beta and gamma oscillations in the cortical–basal ganglia network, thereby stabilizing the normal function of the network. However, STN‐DBS in TS might interfere with the physiology inhibition effect of cortex by interfering with the hyper‐direct pathway to STN from cortex. In the clinical evaluation, we also found the GPi‐DBS is more superior in controlling the obsession in TS patients. The STN‐DBS might lead to impulse control dysfunction such as gambling and hypersexuality in PD (Merola et al., 2017), which might also relate to the compromised physiology inhibition function.

### 4.5 Limitations

Due to the small sample size of this study, it is not reliable to evaluate the relationship between the oscillatory activities and the severity of tics. Hence, it is difficult to determine whether stimulation‐modulated high beta and gamma oscillations are correlated with symptom. However, the aim of the present study was conducted to identify the different effect of GPi and STN‐DBS on the circuits. Consistent STN and GPi‐DBS‐modulated oscillatory activities were seen in individual cases and at the group level, combined with the clinical effect that all patients chose GPi. The hypothesis seems reasonable. The other limitations are that we can only observe the high gamma in the STN when the tics appeared, and the high beta does not change when the tics appeared; we hypothesize that the high beta might reflect a sustained inhibition status which is elevated in TS. We want to conduct the further experiments focusing on the difference between the TS and other movement disorders in the STN to clarify the above questions, but we could not conduct more STN‐DBS in TS due to the limited clinical effect. We also analyzed the LFP change under the stimulation and found STN stimulation (1 V 130 Hz 60 µs) significantly attenuated the high beta and low gamma oscillation power in GPi, but GPi stimulation (1 V 130 Hz 60 µs) do not influence the STN‐LFP, which might relate to the STN‐GPi circuits anatomy. But when the voltage is higher, more complicated phenomenon was observed, since more nuclei and tracts were influenced. Considering the relatively close distance between the two targets, we do not include this part in the result and compare the poststimulation effect. As far as we know, this is the first work on STN‐LFP and STN‐GPi circuit in TS. We feel that with the data available to us, we have generated important information on issues relating to the TS. The findings might be important for the further studies.

## 5 CONCLUSION

**Figure 4:**
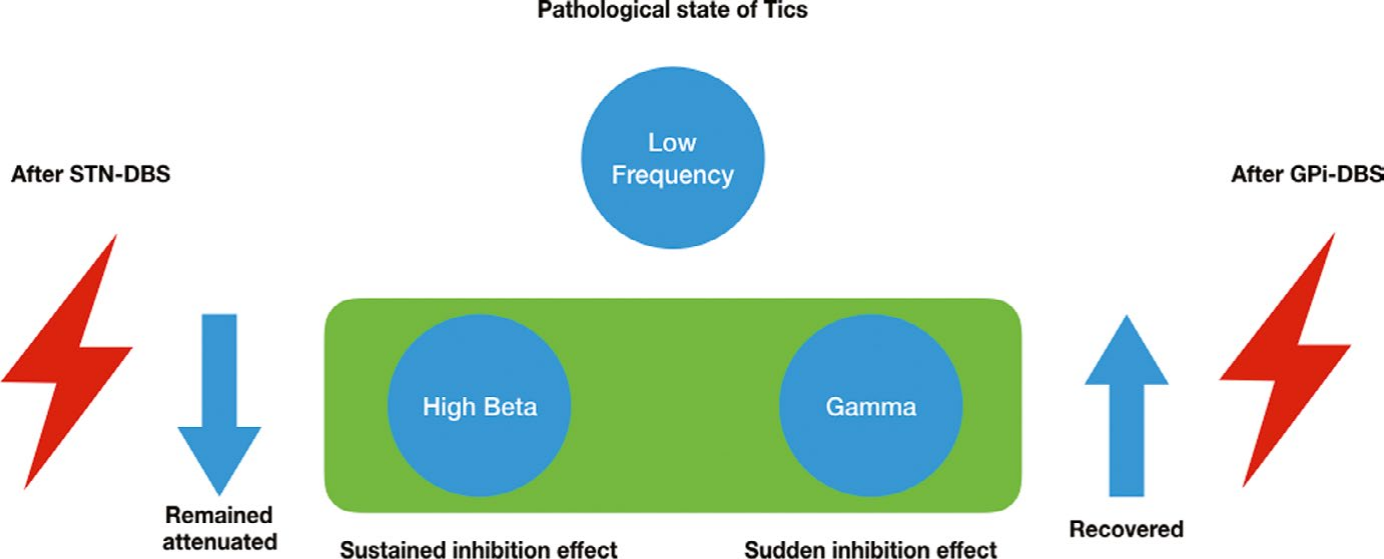
Schematic illustration of the possible mechanism of the different effect of globus pallidus interna‐deep brain stimulation (GPi‐DBS) and subthalamic nucleus (STN)‐DBS for TS. The low‐frequency oscillation in STN and GPi implicates the pathological state of tics. The high beta oscillation underlines a compensatory sustained inhibition effect, and the gamma oscillation underlines a sudden inhibition effect. The high beta and gamma oscillations in STN and GPi remained attenuated after STN‐DBS, while recovering after GPi‐DBS

The LFP in TS represents more complicated functions than PD because the TS involves the psychologic factors. High beta and gamma oscillations in TS may have physiological function in resisting tics in TS. The physiology inhibition function is interfered by the STN‐DBS but not GPi‐DBS which might relate to the limited clinical effect of STN‐DBS (Figure 4), which implies more circuit feedback compensation function are needed to be considered in the development of intelligent neuromodulation for neuropsychiatric diseases like TS.

## CONFLICT OF INTEREST

We wish to confirm that there are no known conflicts of interest associated with this publication and there has been no significant financial support for this work that could have influenced its outcome. We confirm that the manuscript has been read and approved by all named authors and that there are no other persons who satisfied the criteria for authorship is missed. We further confirm that the order of authors listed in the manuscript has been approved by all of us. We confirm that we have given due consideration to the protection of intellectual property associated with this work and that there are no impediments to publication, including the timing of publication, with respect to intellectual property. In so doing, we confirm that we have followed the regulations of our institutions concerning intellectual property. We further confirm that any aspect of the work covered in this manuscript that has involved either experiment animals or human patients has been conducted with the ethical approval of all relevant bodies and that such approvals are acknowledged within the manuscript. We understand that the corresponding author is the sole contact for the editorial process (including editorial manager and direct communications with the office). He/she is responsible for communicating with the other authors about progress, submissions of revisions, and final approval of proofs. We confirm that we have provided a current, correct email address which is accessible by the corresponding author and which has been configured to accept email from zjguo65@163.com.

## AUTHOR CONTRIBUTION

Guan‐Yu Zhu conducted the experiments, collected MRI and clinical information, analyzed and interpreted the data, and drafted the manuscript. Xin‐Yi Geng conducted the experiment, collected the electrophysiological data, analyzed and interpreted the data, and drafted the manuscript. Rui‐Li Zhang conducted the experiments and helped to collect the data, and analyzed the data. Ying‐Chuan Chen and Yu‐Ye Liu helped to evaluate the effects of postoperative stimulation. Jian‐Guo Zhang supervised the design of the experiments, performed the surgery for DBS and the clinical evaluation, and revised the manuscript. Shou‐Yan Wang supervised the design of experiment and data analyses, and revised the manuscript.

## Data Availability

Data were made available to all interested researchers upon request.
