# Supplementary figures and images for: Deep brain stimulation modulates pallidal and subthalamic neural oscillations in Tourette's syndrome
Source: Brain Behav. 2019 Oct 24;9(12):e01450. doi: 10.1002/brb3.1450 (PMC6908859; doi:10.1002/brb3.1450)

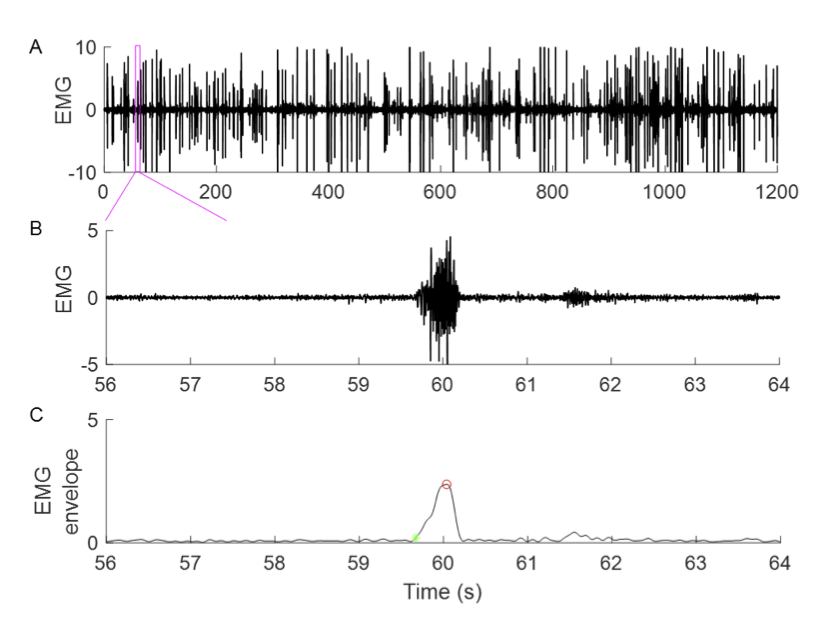

Supplement: Supplementary file 1 [file BRB3-9-e01450-s001.tiff]

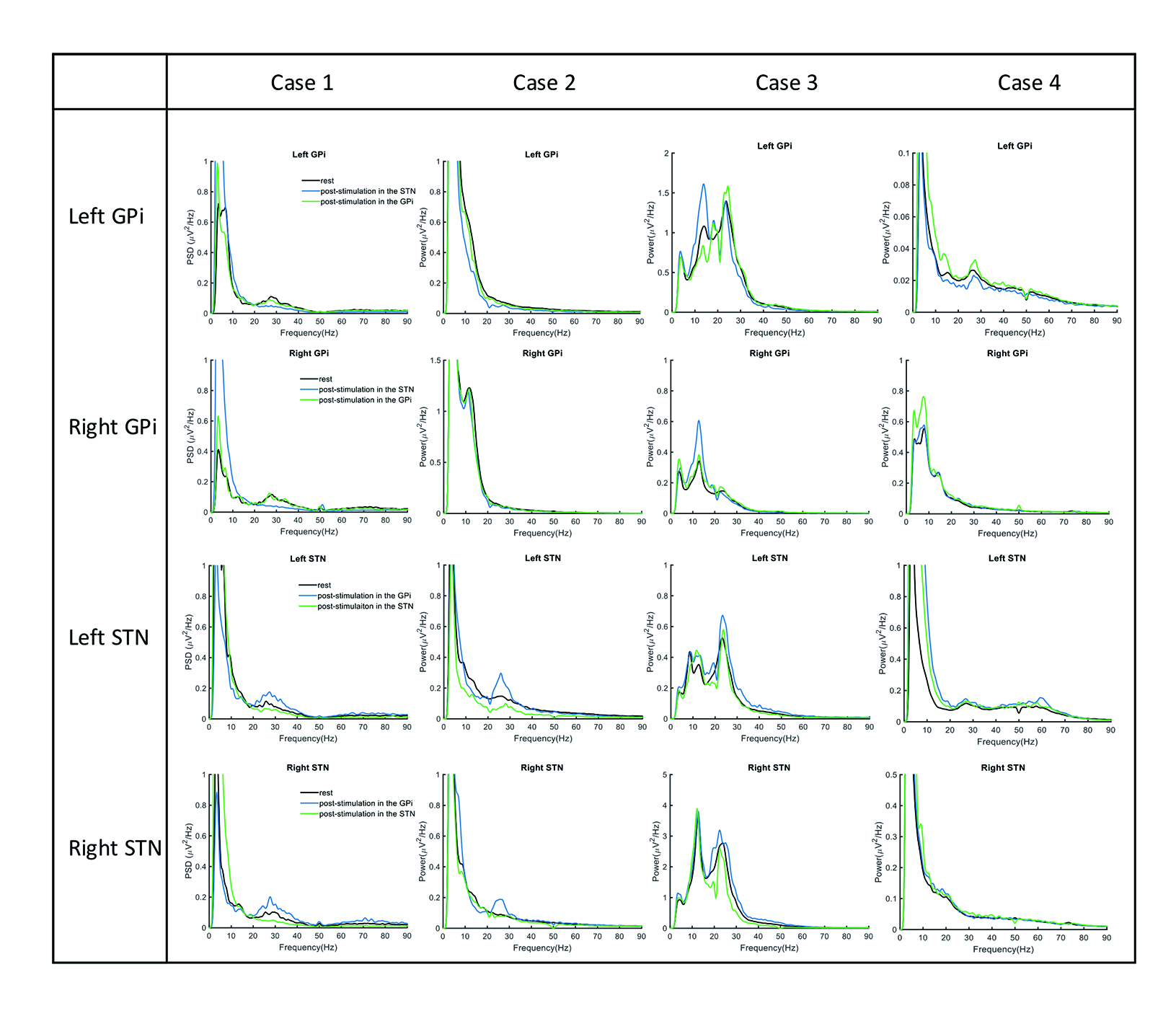

Supplement: Supplementary file 2 [file BRB3-9-e01450-s002.tiff]
